# Navigating complexity: a conceptual framework for simulation interventions

**DOI:** 10.1186/s41077-025-00366-y

**Published:** 2025-07-06

**Authors:** Anders L. Schram, Tine Brink Henriksen, Helle Terkildsen Maindal, Victoria Brazil

**Affiliations:** 1 Department of Clinical Medicine, MidtSim, Aarhus University, Hedeager 5, Aarhus, 8200 Denmark; 2 Department of Clinical Medicine, Aarhus University, Aarhus, Denmark; 3 Department of Public Health, Aarhus University, Aarhus, Denmark; 4 Faculty of Health Sciences and Medicine, Bond University, Gold Coast, QLD Australia

## Abstract

**Background:**

Healthcare systems are inherently complex, shaped by dynamic interactions and interdependencies rather than rigid structures. Simulation-based training interventions must embrace this complexity. Complex Adaptive Systems and Resilient Healthcare provide complementary theoretical frameworks for understanding how healthcare systems can respond to internal and external needs while maintaining adaptability and functionality. Incorporating concepts from Complex Adaptive Systems and Resilient Healthcare into simulation-based interventions increases the likelihood of their success within contemporary healthcare systems. A focus on adaptability, continuous learning, and system-wide resilience is necessary for healthcare improvement, and simulation interventions can help develop and reinforce these capabilities. In this article, we argue that simulation must be reimagined to reflect the realities of complex healthcare systems and propose a conceptual framework to support this shift.

**Main body:**

We propose a three-component conceptual framework for simulation practitioners seeking to design and deliver interventions that embrace complexity: (1) *Problem identification*, (2) *simulation design*, and (3) *evaluation strategies*. The three components function across organizational levels, supporting a dynamic and adaptive approach to addressing healthcare system challenges. By integrating Complex Adaptive Systems and Resilient Healthcare principles, simulation-based interventions can foster a complexity-aware mindset, enabling healthcare professionals and organizations to anticipate, respond to, and recover from challenges more effectively.

To illustrate this framework, we introduce three vignettes demonstrating how simulation-based interventions may benefit at different levels within healthcare systems. The vignettes illustrate how challenges at the institutional, departmental, and individual levels can be identified and addressed effectively by simulation-based interventions.

**Conclusion:**

Simulation interventions can strengthen healthcare systems by supporting organizational learning and embedding principles from complexity science and resilience thinking. This requires reimagining simulation not as isolated training events but as complex interventions that operate across levels and respond to dynamic system needs. By adopting this systems-based approach, simulation practitioners, healthcare leaders, and policymakers can better align simulation with real-world conditions — bridging theory and practice while fostering more adaptive and resilient care.

## Introduction

Healthcare systems must continuously adapt to shifting demands, disruptions, and evolving interdependencies [1, 2]. Unlike mechanistic systems with predictable outcomes, healthcare functions as a dynamic, interdependent network [3, 4]. Simulation is increasingly recognized for its role in strengthening healthcare systems, extending beyond individual training to address system challenges [5]. However, despite growing interest [6, 7], system-focused simulation interventions often lack consistency in design and application [8]. Despite the growing relevance of complexity science, many healthcare practitioners remain unaware of how concepts from Complex Adaptive Systems and Resilient Healthcare could enhance simulation’s contribution to system-wide learning and resilience [9].

To bridge this gap, we aim to propose a framework that aligns simulation interventions with Complex Adaptive Systems and Resilient Healthcare, enabling a more adaptive and integrated approach to healthcare challenges. Through three vignettes, we illustrate how simulation can enhance resilience and system-level learning in complex healthcare environments.

### Complexity in healthcare: a theoretical perspective

Healthcare is inherently complex, characterized by interdependencies and unpredictable interactions [9–11]. Success depends on individual expertise and the system’s ability to adapt to shifting constraints [12]. Complex Adaptive Systems theory has consistently provided insights into healthcare functioning beyond rigid structures. The theory views healthcare systems as networks of interacting agents whose behaviours are shaped by context and history [13, 14]. Outcomes emerge through these interactions and cannot be fully understood by analyzing individual components in isolation. Rather than focusing on control, Complex Adaptive Systems theory emphasizes understanding and adaptation through continuous feedback and local decision-making [13, 14]. Yet, many organizations still assume linear, reductionist approaches that prioritize predictable workflows over adaptability [15, 16]. This misalignment hinders attempts to implement complexity-based strategies [17, 18]. Healthcare involves multiple interacting elements, such as clinicians, patients, technology, and policies. These factors continuously shape system dynamics, influencing how teams respond to challenges, allocate resources, and maintain care quality [19–21].

Recognizing this need for adaptability, Resilient Healthcare builds on Complex Adaptive Systems principles, emphasizing flexibility over rigid error-reduction strategies to support quality and safety [1]. Resilient Healthcare acknowledges that variability in clinical work is inevitable, and that resilience stems from the ability to anticipate, respond to, and recover from disruptions [22]. Instead of treating variability as a risk, Resilient Healthcare highlights its role in maintaining safe and effective healthcare [22]. Understanding everyday work, including “workarounds,” guides improvement. As Nason notes, embracing complexity requires recognizing its characteristics and developing a mindset attuned to how complex systems behave [9]. In healthcare leadership, this mindset rarely arises from awareness alone. It tends to evolve through sustained engagement with system-level challenges and practical experience with clinical work’s unpredictable and adaptive nature [9]. Table 1 presents selected examples of influential resources on Complex Adaptive Systems and Resilient Healthcare, each with a brief description.

**Table 1:** Key resources on complex adaptive systems and resilient healthcare

Topic	Reference (first athor (year): title)	Key insights
CAS	Braithwaite et al. (2017) [18]: *Complexity science in healthcare*	Explores the application of complexity science to healthcare, highlighting how understanding CAS can improve healthcare delivery and policy
CAS	Ellis (2011) [13]: *Complex adaptive systems (CAS): an overview of key elements, characteristics and application to management theory*	Provides an overview of CAS elements and characteristics, discussing their application to management theory, including insights into evolutionary change processes in healthcare
CAS	McNab et al. (2020) [19]: *Development and application of ‘systems thinking’ principles for quality improvement*	Discusses the development and application of systems thinking principles, rooted in CAS theory, for enhancing quality improvement initiatives in healthcare
CAS	Pype et al. (2018) [14]: *Healthcare teams as complex adaptive systems*	Explores how healthcare teams function as CAS by analyzing team members’ perceptions of interpersonal interactions, offering insights into team behavior and dynamics
CAS	Swanson et al. (2012) [15]: *Rethinking health systems strengthening*	Discusses the application of systems thinking tools and strategies to achieve transformational change in health systems, emphasizing the importance of understanding CAS dynamics
RHC	Iflaifel et al. (2020) [22]: *Resilient health care: a systematic review*	Reviews different conceptualizations of resilience in healthcare, research methodologies, and key factors influencing resilience development
RHC	Lyng et al. (2021) [1]: *Balancing adaptation and innovation for resilience in healthcare*	Examines the balance between adaptation and innovation in healthcare resilience, synthesizing various narratives
RHC	Woods (2015): *Four concepts for resilience and the implications for the future of resilience engineering*	Defines four key resilience concepts and their relevance to healthcare systems
Other viewpoints	Braithwaite et al. (2018) [23]: *When complexity science meets implementation science: a theoretical and empirical analysis of systems change*	Examines the intersection of complexity science and implementation science to drive systemic change, providing both theoretical insights and practical applications
Other viewpoints	Nason (2023) [9]: *Challenges of implementing complexity in healthcare*	Explores why complexity thinking is not widely adopted in healthcare, presenting a framework for evaluating complexity acceptance
Other viewpoints	Plsek (2001) [10]: *Complexity science: the challenge of complexity in health care*	Discusses various perspectives on complexity in healthcare, highlighting adaptive strategies and systemic interdependencies

*CAS* Complex Adaptive Systems, *RHC* Resilient Healthcare

### Simulation as an intervention within complex systems

Aligned with principles from Complex Adaptive Systems and Resilient Healthcare, simulation can support the exploration of system variability, test adaptive strategies, and strengthen system-wide learning and adaptive capacity [13, 14, 22]. It offers opportunities to explore everyday work and gather insights from practitioners — through thoughtful debriefing — about “work as done,” including perceived resilience and system weaknesses [24]. Through scenarios that recreate common challenges, practitioners can refine their ability to anticipate, respond to, and recover from disruptions, reinforcing resilience at individual, team, and system levels [22]. Moreover, simulation enables organizations to identify misalignments between individual competence and system design, such as missing equipment or communication breakdowns, which may hinder performance despite technical proficiency [25, 26]. In this way, simulation extends beyond skill acquisition to actively foster a complexity-aware mindset and facilitate system-level sense-making and adaptation [1, 8, 27].

### A conceptual framework for simulation embracing complexity

We propose a conceptual framework to systematically integrate Complex Adaptive Systems and Resilient Healthcare into simulation interventions. We aim to bridge theory and practice to ensure simulation interventions better capture the realities of healthcare systems [23]. In developing the framework, we are informed by empirical evidence and the authors’ collective experience. AS specializes in simulation implementation and evaluation; TBH brings extensive clinical experience, including teaching across diverse healthcare settings; HTM is a public health professor with expertise in complex healthcare interventions and implementation science; and VB, professor of simulation education, has led the international academic conversation on translational simulation [7, 27], focused on simulation to enhance team performance and systems improvement. By combining these perspectives, our framework offers a structured approach for simulation practitioners, quality improvement practitioners, and policymakers to develop simulation-based interventions that strengthen system-wide improvements.

To effectively tackle healthcare challenges, we recommend structuring simulation interventions around three components as follows: (1) *Problem identification*, (2) *simulation design*, and (3) *evaluation strategies* (Table 2). These components were selected because they reflect key phases in developing effective simulation-based interventions and align with established models for designing and evaluating complex interventions in healthcare [28–30]. Firstly, *problem identification* entails recognizing and clearly defining a specific challenge within the broader complexity of the healthcare system. Secondly, *simulation design* focuses on developing and implementing customized strategies to address this problem. Thirdly, *evaluation strategies* involve examining how simulation interventions contribute to meaningful change in practice, with attention to effects that may unfold across different organizational levels. As shown in Table 2, such interventions can influence structures, processes, and behaviors at the institutional, departmental/team, and individual levels [31].

**Table 2:** Examples of problems identification, simulation design, and evaluation strategies across organizational levels

		Components in simulation interventions		
		Problem identification	Simulation design	Evaluation strategies
Organizational levels	Institutional	Workflow disruptions during hospital relocation	Walk-throughs, tabletop exercises, and in situ simulations to test layout and workflows	Staff adaptation, workflow efficiency, perceived preparedness
		Workflow inefficiencies due to outdated EMR systems	Simulation of novel EMR integration and digital workflows	Care process efficiency, staff experience, unintended outcomes
	Departmental/team	Miscommunication during handovers causing birth asphyxia	Interdisciplinary team training using ISBAR and escalation scenarios	Birth asphyxia rates, teamwork effectiveness, staff reflections
		Challenges in timely escalation of care for deteriorating patients	Role clarification and structured team training	Time to intervention, quality of decision-making
	Individual	Low first-pass success in intubation	Airway emergency simulation using manikins	Task performance, protocol adherence, confidence
		Hesitation in equipment use during emergencies	Hands-on task training using checklists	Task completion time, usability, learning outcomes

*EMR* electronic medical records, *SBAR* situation, background, assessment, and recommendation

**Fig. 1:**
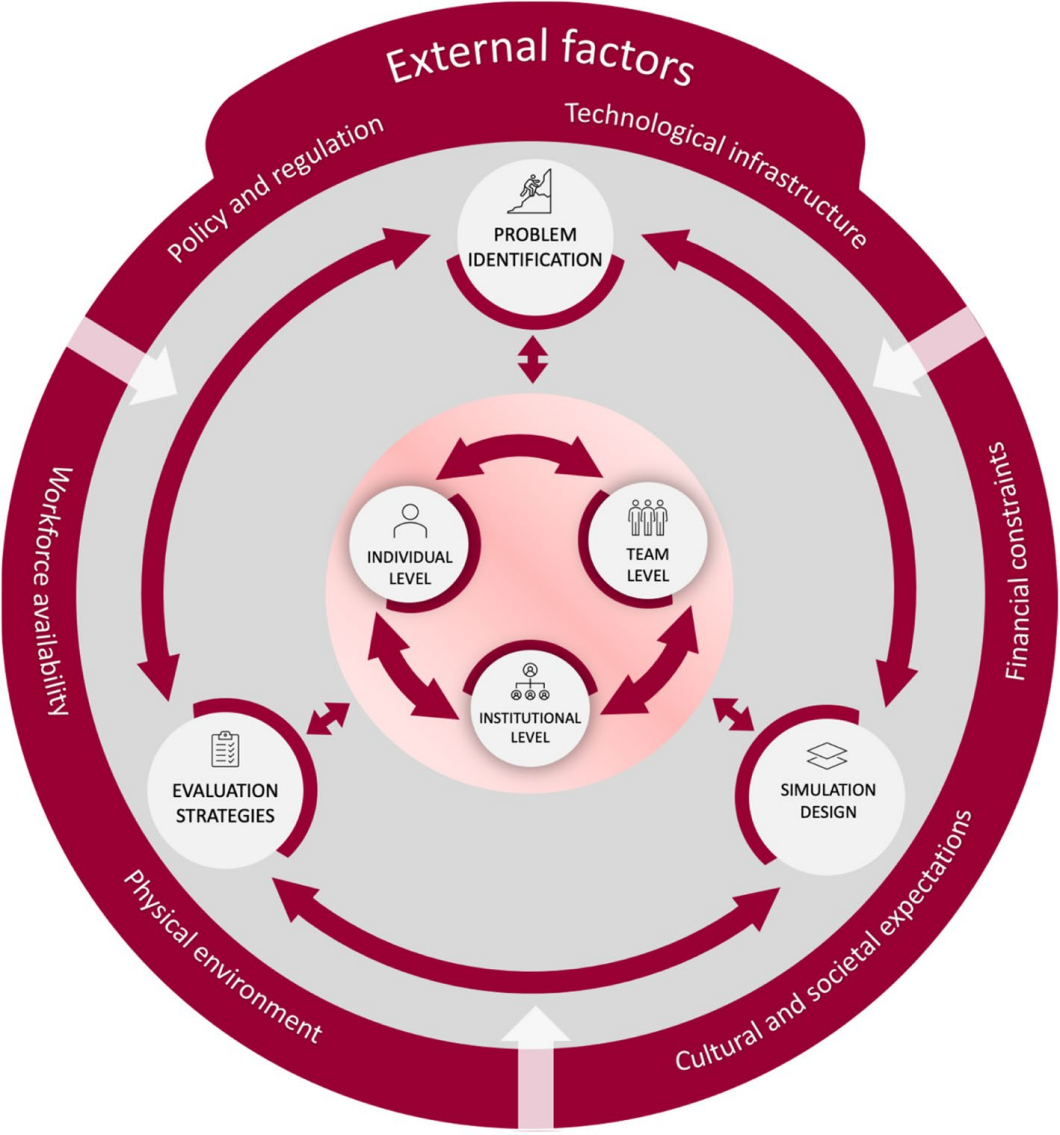
Factors affecting simulation interventions

Table 2 provides a structured overview of examples comprising components in simulation interventions, but the complexity lies in the numerous variables and their dynamic interactions. Thus, Fig. 1 presents a conceptual framework illustrating the interaction between *problem identification*, *simulation design*, and *evaluation strategies* across organizational levels. The framework also points to external factors that can either support or challenge simulation efforts, such as policy conditions or constraints in infrastructure. These broader influences help shape how simulation unfolds in practice and connects with existing institutional, team, and individual dynamics. Similar macro-level influences are frequently described in implementation science and health systems research [30].

As an example, outdated electronic medical record (EMR) systems may create workflow inefficiencies, leading to delays in patient care (*problem identification*) [32] (Table 2). In response, hospitals can introduce new EMR systems and simulate their integration (*simulation design*) [33]. However, delays in patient care are not solely influenced by technological issues. At a departmental level, for instance, teamwork failures could contribute to these delays (*additional problems*) [34], while at the individual level, mishandling of medical equipment may also play a role (*additional problems*) [35] (Table 2). A holistic, multi-level approach is needed to address these challenges effectively because multiple factors contribute to patient care delays. This dynamic interplay extends across *problem identification, simulation design*, and *evaluation strategies*. For instance, in addition to simulating system integration to improve workflow inefficiencies caused by outdated EMR systems, the best results may be achieved by strengthening team communication in general and ensuring protocol adherence (*additional simulation design elements*).

Beyond improving workflows, simulating the integration of a new EMR system may also generate outcomes at other evaluation levels (Table 2 and Fig. 1). While such interventions primarily target system-level improvements, like staff adaptation and cost efficiency, they may also influence team and individual dynamics. For instance, integrating a new EMR system can enhance interprofessional collaboration, clarify roles in digital workflows, and increase user confidence. However, unintended consequences may also emerge at different levels, including increased documentation burden, inefficient communication patterns, or reduced engagement due to resistance to change [36, 37]. Thus, evaluating complex simulation interventions requires careful interpretation and a recognition of their multifaceted impact [5, 29]. Selecting appropriate assessment and exploration tools and critically interpreting effects are essential. We suggest that quantitative and qualitative measures should be integrated to provide a more nuanced and comprehensive understanding of the impact of complex simulation interventions [29]. Such an approach contributes to understanding why and under what conditions an intervention brings about positive change [38]. It also shifts the purpose of evaluation from merely proving success to improving future simulation interventions [39]. This distinction is critical. Outcome-based evaluations often prioritize demonstrating positive results, overlooking unintended or negative consequences [39]. Focusing on continuous improvement, rather than solely proving effectiveness, allows for a deeper understanding of the broader implications of these interventions, particularly their adaptability and relevance in diverse healthcare contexts [30, 40].

Lastly, as illustrated in Fig. 1, external factors are crucial in shaping simulation interventions’ feasibility and long-term impact [5, 41]. Supportive conditions, such as institutional investment in infrastructure or prioritization of digital integration, can help enable the successful implementation of new EMR systems [21]. In contrast, restrictive policies, including budget reductions or shifting organizational priorities, may hinder deployment efforts. These external pressures can reduce staff engagement and interfere with how workflows adapt over time, making it harder to sustain the intervention [42]. Acknowledging and addressing such factors are essential to embedding simulation interventions in complex healthcare systems [15].

### Complex simulation interventions in practice

We present three hypothetical vignettes inspired by the author’s experiences and the existing literature. These vignettes illustrate the practical application of our framework and demonstrate how simulation interventions can systematically address complex healthcare challenges. The vignettes illustrate how structured *simulation design* can be applied in practice, guided by meticulous *problem identification*. Each case highlights the use of structured methodologies to first identify a key problem within the system before designing targeted simulation interventions with a proposed *evaluation strategy* approach.

#### Vignette 1: Hospital relocation (institutional level)

##### Problem identification

Hospital A is being redeveloped. A new building is planned with many modern design features that are absent from the old hospital. A stakeholder analysis was conducted to identify key risks, including workflow disruptions, communication breakdowns, and staff uncertainty about new processes. Key concerns included difficulties adapting to new spatial layouts, unfamiliar equipment placement, and potential patient flow bottlenecks, all of which posed risks to clinical efficiency. Staff dissatisfaction and resistance to relocation further underscored the need for structured interventions to support adaptation. According to existing literature, hospital transitions are associated with increased stress, higher sick leave, and reduced efficiency among healthcare professionals [43]. Hospital relocations pose logistical and operational challenges that require structured preparation to prevent disruptions to patient care and staff workflows [43].

##### Simulation design and evaluation strategies

It was recognized that traditional onboarding approaches, such as orientation sessions or passive informational materials, often fail to prepare staff for real-world complexities in new environments [43, 44]. Thus, a translational simulation strategy was developed to test new hospital workflows, refine spatial organization, and identify inefficiencies before full implementation [43]. A three-phase approach was used as follows:

1. Workflow testing simulations: Staff navigated the new hospital environment through “walk-through” and tabletop simulations, identified inefficiencies in patient flow, and refined processes to prevent bottlenecks.
2. Emergency preparedness simulations: In situ simulations were used to assess the functionality of alarm systems, clarity of emergency routes, and adequacy of security protocols. Feedback from these sessions informed adjustments to infrastructure and procedures to enhance emergency preparedness at a system level.
3. Critical event response testing: Simulations of time-critical situations were conducted to evaluate how new workflows and spatial layouts supported coordinated responses. Observations from these simulations revealed latent safety threats and informed system refinements to better support decision-making under pressure.

Successful implementation required early stakeholder involvement, clear training objectives, and integration into existing workflows [43]. Leadership commitment and organizational readiness were essential for long-term sustainability. To determine the intervention’s long-term impact on hospital operations and patient safety, qualitative interviews on staff adaptation and workflow efficiency measurements can be used to evaluate impacts.

#### Vignette 2: Birth asphyxia (department level)

##### Problem identification

A root cause analysis, incorporating retrospective case reviews and structured staff interviews, identified factors contributing to birth asphyxia cases in an obstetric department [45]. These factors included ineffective clinical handovers and differing professional perspectives, leading to delays in decision-making. Aligning with current research in maternity care, key findings highlighted miscommunication during handovers and inconsistencies in how midwives, obstetricians, pediatricians, and anesthesiologists interpreted patient information, which led to impaired team coordination and delays in neonatal resuscitation [46, 47].

##### Simulation design and evaluation strategies

Interdisciplinary simulation was used as an approach to improve delivery room communication [48]. *Simulation design* elements included the following:

1. Embedding standardized communication frameworks, such as identity, situation, background, assessment, and recommendation (ISBAR), to support clinical handover, including clear patient identification and location
2. Team participation in training to enhance role clarity, early recognition of clinical red flags, and collaborative situational awareness
3. Process testing simulations to assess workflow efficiency and optimize escalation procedures. Successful implementation required clear communication protocols, interdisciplinary collaboration and institutional support to ensure sustainability.

The *Evaluation strategy* included pre- and post-intervention birth asphyxia rate comparisons, qualitative feedback from healthcare professionals, and observational assessments of teamwork and communication improvements during neonatal resuscitations.

#### Vignette 3: Airway management (individual level)

##### Problem identification

An emergency department audit of airway management revealed performance below accepted benchmarks for “first-pass success” in endotracheal intubation [49]. A process mapping approach was used to systematically identify inefficiencies in airway management [50]. The process mapping was combined with simulation-primed qualitative inquiry [51] in which staff participated in intubation scenarios followed by interviews to understand their decision-making and actions. This traced each step of the clinical workflow to pinpoint areas where deviations from standard protocols were necessary. Findings revealed that standard clinical protocols often failed to accommodate patient-specific variations, particularly in complex cases requiring deviations from standard procedures [40].

##### Simulation design and evaluation strategies

Having already utilized simulation to explore airway issues, the next phase involved simulation used as a “test bed” for improvement. Simulations of variations in airway emergencies, using specifically prepared manikins, allowed teams to test alternative intubation techniques when standard procedures prove ineffective [52]. The difficult airway kit was redesigned based on staff feedback from the simulations. Successful implementation resulted from leadership support for staff to participate in iterative simulations. The evaluation included time-to-task completion metrics in simulation, departmental first-pass intubation success rates, and self-assessment tools to measure participants’ confidence and preparedness in managing complex airway scenarios.

## Conclusion

The healthcare simulation community should adopt key principles from Complex Adaptive Systems and Resilient Healthcare to effectively support healthcare improvement. This requires rethinking how simulation is developed and implemented as a complex intervention, using approaches that may be unfamiliar within traditional simulation-based education. Evaluating the impact of these simulation-based improvements also demands a broader perspective, considering multifaceted and interconnected outcomes. We encourage simulation practitioners, healthcare leaders, and policymakers to adopt this systems-based approach when designing and implementing simulation interventions, ensuring they align with real-world healthcare environments’ complexities and optimize their impact.

## Data Availability

No datasets were generated or analysed during the current study.
